# Potentialities and Challenges of mRNA Vaccine in Cancer Immunotherapy

**DOI:** 10.3389/fimmu.2022.923647

**Published:** 2022-05-26

**Authors:** Li-Juan Duan, Qian Wang, Cuilian Zhang, Dong-Xiao Yang, Xu-Yao Zhang

**Affiliations:** ^1^ Medical School, Huanghe Science and Technology College, Zhengzhou, China; ^2^ Reproductive Medicine Center, Henan Provincial People’s Hospital, People’s Hospital of Zhengzhou University, Zhengzhou, China

**Keywords:** mRNA, cancer vaccine, immunotherapy, efficient delivery, optimization, strategies

## Abstract

Immunotherapy has become the breakthrough strategies for treatment of cancer in recent years. The application of messenger RNA in cancer immunotherapy is gaining tremendous popularity as mRNA can function as an effective vector for the delivery of therapeutic antibodies on immune targets. The high efficacy, decreased toxicity, rapid manufacturing and safe administration of mRNA vaccines have great advantages over conventional vaccines. The unprecedent success of mRNA vaccines against infection has proved its effectiveness. However, the instability and inefficient delivery of mRNA has cast a shadow on the wide application of this approach. In the past decades, modifications on mRNA structure and delivery methods have been made to solve these questions. This review summarizes recent advancements of mRNA vaccines in cancer immunotherapy and the existing challenges for its clinical application, providing insights on the future optimization of mRNA vaccines for the successful treatment of cancer.

## Introduction

Cancer is one of the most lethal diseases in the world. In the recent decades, achievements in the understanding of the immune system have shed light on the treatment of cancer by immunotherapies (1). Various immune cells are able to identify antigens on the surface of cancer cells and interact with the antigenetic peptides to destroy cancer cells. Therefore, immunotherapies including immune cell-based cancer vaccines were considered as promising approaches to cure cancer. Cancer vaccines have drawn great attention since the clinical application of several novel cancer vaccines such as immune cell-based vaccines, viral vector-based vaccines and RNA or DNA-based vaccines (2, 3). Among these, mRNA-based cancer vaccines demonstrate exceptional advantages in comparison with the other types of vaccines (4). First, unlike virus-based vaccines which can be infectious in some circumstances, mRNA-based vaccines are safer and free of contamination. Second, once delivered into the cells, the genetic information of antigen carried by mRNA can be translated into protein very rapidly. Third, mRNA-based cancer vaccines can stimulate immune response and overcome vaccine resistance that is often observed in conventional chemotherapies. In addition, mRNA-based cancer vaccines encode the cancer antigens in full-length and thus can overcome the restrictions of human leukocyte antigen to activate a broader immune response. Last but not least, mRNA-based cancer vaccines are free of mutations since mRNA can’t insert into the chromosome. Given the advantages of mRNA vaccines over conventional cancer vaccines, this novel immunotherapy has become the hotspot of research for the development of new generation cancer therapies. However, the expected substantial application of mRNA-based cancer vaccines was not seen due to several problems regarding the instability of mRNA, immunogenicity and the inefficiency of *in vivo* delivery.

Research on mRNA-based vaccines have focused on overcoming the instability and the delivery of mRNA. One strategy is to use synthetic mRNA or modified mRNA analogs, which can enhance the stability and protein expression of mRNA. For example, circular RNA is used to avoid the detection of pathogen-associated pattern receptors; self-amplifying RNA greatly increased the expression level of proteins (5). Another strategy is to modify the *in vivo* delivery methods of RNA vaccines to achieve higher efficiency. For instance, in addition to electroporation, novel vectors such as polyplexes, lipid nanoparticles, peptides and cationic nanoemulsions have been used for the delivery of RNA vaccines (6). The success of RNA-based vaccines for the COVID19 pandemic has witnessed the significant progress made on the clinical application of mRNA vaccines and the increasing necessity of developing novel RNA vaccines for treatment of diseases (7).

In this review, we will focus on the most recent progress that have been made on the stability and *in vivo* delivery of mRNA-based vaccines for the treatment of cancer and discuss the existing challenges on the current clinical application of mRNA-based immunotherapies, hoping to accelerate the clinical application of mRNA-based cancer immunotherapy.

## Evolution of mRNA-based Vaccines in Cancer Immunotherapy

The first introduction of mRNA to activate immune response *in vivo* could be dated to the year 1993, when Martinon and co-workers constructed a liposome-mRNA expressing influenza hemagglutinin that activated CD8^+^ T cell responses for the detection and lysing of virus-infected cells (8). In 1995, mRNA construct expressing cancer embryonic antigen was reported to induce the generation of antibodies in mice, demonstrating the potential of mRNA vaccines in cancer therapies (9, 10). Over the past decades, mRNA vaccines quickly became the spotlight of cancer immunotherapy due to their abilities to provide safe vaccination, to improve antigen expression and to avoid gene integration. The earliest mRNA vaccines in cancer immunotherapy normally use RNA virus genomes, which have shown good efficacy against viral cancer in mouse models (11). Later, a liposome-mRNA vaccine was developed and its ability to induce cognate cytotoxic T cells has resulted in the destroy of melanoma cancer cells (12). These mRNA-based vaccines have demonstrated great advantage over conventional vaccines and thus have been established as novel strategies for cancer therapies. Since then, more and more kinds of mRNA-based vaccines are developed for treatment of cancer.

To date, replicating modified mRNA, unmodified mRNA, and virus-derived mRNA are the three main types of mRNA vaccines in cancer immunotherapy (1). The basic structure of non-replicating mRNA consists of an open reading frame flanked by a 5′-prime region and a 3′-prime untranslated region (UTR), a 5′ cap structure and a 3′ poly (A) tails (13). The ORF of mRNA encodes the sequences of the target antigen. The target antigen will be translated into proteins once mRNA is transited to the cytosol of the cell. The expressed antigen will further be post-translationally modified and fold into a full-functional protein whereas the remaining mRNA will be quickly degraded to reduce potential toxicity. However, the rapid degradation of naked mRNA also dramatically affects its stability. In addition, the intrinsic immunogenicity of mRNA further promotes the rapid degradation of mRNA, resulting in decreased expression levels of antigen. Moreover, the inefficient *in vivo* delivery of mRNA-based vaccines will reduce the protein expression of mRNA. These disadvantages have greatly limited the clinical application of mRNA vaccines in cancer immunotherapy. We will discuss the limitations and highlight the current state-of-the-art strategies to overcome these limitations in the next section.

## Strategies to Improve the Stability of mRNA-based Cancer Vaccines

Previous studies have found that the inadequate methylation of mRNA, impurity and immunogenicity could lead to low efficiency of mRNA translation (14, 15). A better understanding of the structure of mRNA has provided new strategies to improve the stability and the translation efficiency of mRNA.

**Figure 1:**
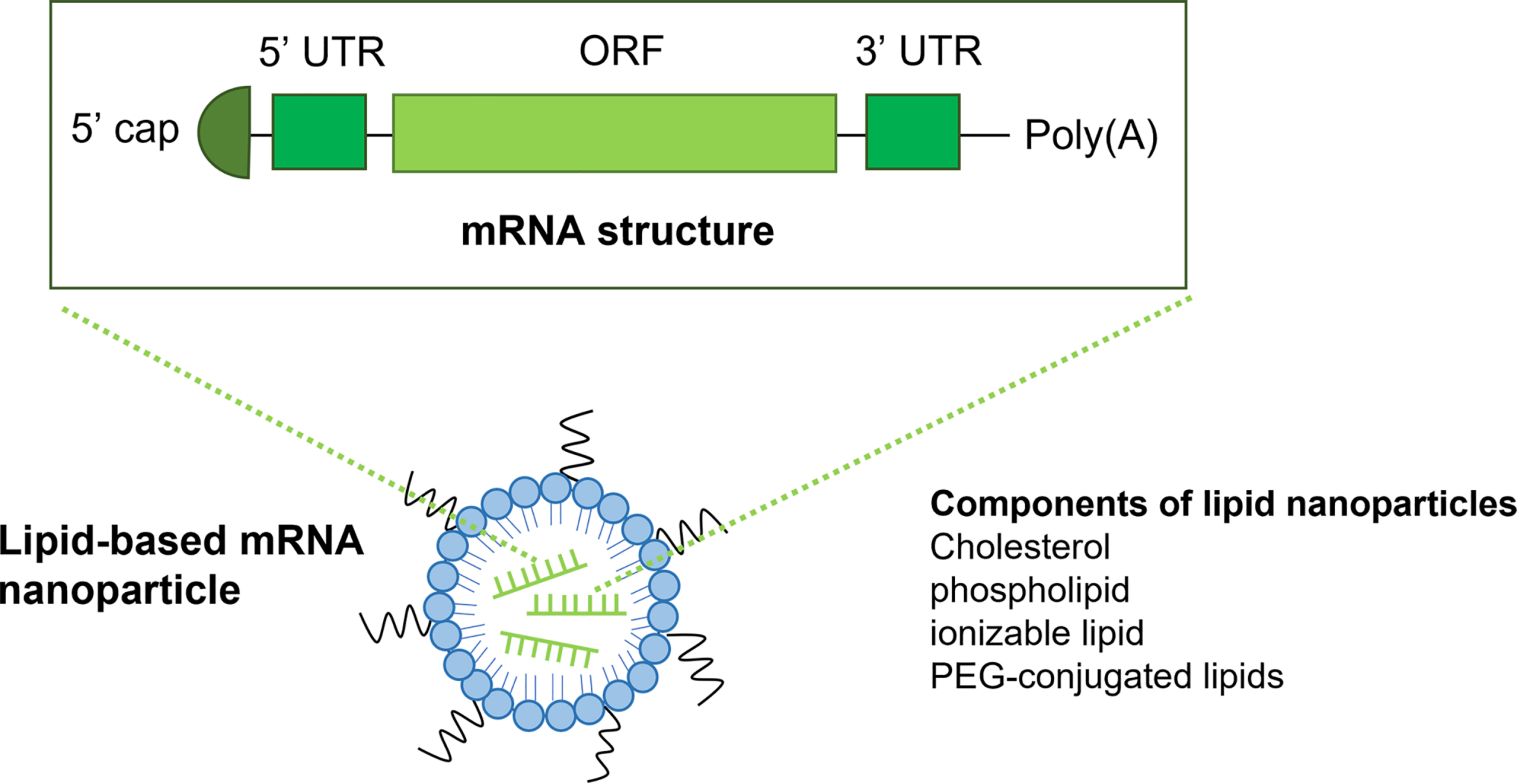
The structure of mRNA and lipid-based nanoparticles. The structure of mRNA (top) consists of a 5’ cap, a 5’UTR and a 3’ UTR, an open reading frame and a poly(A) tail. Lipid nanoparticles (LNPs) are the most commonly used vectors for the delivery of mRNA vaccines. LNPs often comprise of ionizable lipid, Cholesterol, phospholipid and PEG-conjugated lipids.

mRNA consists of an ORF, a 5′-prime UTR, a 3′-prime UTR, a 5′ cap structure and a 3′ poly (A) tails (**Figure 1**). Firstly, modification of the 5’ cap of mRNA has been utilized to improve stability. There are mainly two types of mRNA capping methods: the *in vitro* post-translational capping enzymatic method that utilizes the Vaccinia virus Capping Enzyme (VCE) and the chemical capping methods (16). These capping methods largely improve mRNA translation but are limited by reverse incorporation. This limitation could be overcome by the development of anti-reverse cap analogs (ARCA), which is methylated at the C3 position. ARCA significantly increases the expression of mRNA. However, the disadvantages of ARCA including de-capping of mRNA, exogeneous motif, and relatively low capping efficiency (60-80%) in contrast to the enzymatic capping method (100%) (17, 18). To increase the capping efficiency and prevent de-capping of ARCA, a novel co-transcriptional cap analog termed CleanCap has been developed, which can generate an unmodified cap structure with high efficiency (>90%) (19).

Secondly, post-translational modifications of mRNA can inhibit the immune recognition of mRNA. Studies have revealed that minimization of mRNA reorganization by the innate immune system could prevent sensing and destruction of mRNA translation, resulting in increased expression (15, 20). For example, removal of the uncapped phosphate of mRNA by phosphatases can effectively avoid immunogenicity. In addition, modifications of mRNA through substitution of natural uridine and cytidine with pseudouridine (Ψ) or 1-methylpseudouridine (m1Ψ) can also effectively avoid immunogenicity and improve stability (21, 22). Pseudouridine is an isomer of uridine in which the nitrogen-carbon glycosidic bond of uridine is replaced by a carbon-carbon bond. N1-Methylpseudouridine is the methylated derivative of pseudouridine and is usually used for mRNA vaccines. Substitution of uridine with pseudouridine (Ψ) or 1-methylpseudouridine (m1Ψ) in mRNA synthesis can reduce cytotoxicity of mRNA vaccines because Ψ/m1Ψ mRNAs trigger low-to-no immune responses to the cells and enhance the expression of tumor antigen (23, 24).

Thirdly, increased purity of RNA and removal of impurities can prevent the degradation of mRNA. During the process of transcription, double stranded RNA impurities are easily formed, which leads to the immunogenicity of mRNA cancer vaccines. Thess and co-workers have shown that the RNA impurities could be removed by optimization of the sequence and purification of RNA by high-pressure liquid chromatography (HPLC), which could result in reduced immunogenicity and improved stability of mRNA vaccines (25). However, HPLC is limited by low yield and high cost. A better way to achieve high purity of mRNA is to use synthetic mRNA (26).

Fourthly, optimization of the ORF of mRNA can improve mRNA translation. Increased GC content in the ORF can be applied to improve stability (27). Methods to increase GC content including codon optimization of ORF and depletion of uridine (28). For example, rare codons in ORF are often substituted by codons with higher tRNA abundance to achieve higher translation rate (29). However, high translation rate sometimes can be harmful, which is the major limitation of codon optimization (30). The reason underlying this observation is that the correctly folding of some proteins may require a low translation rate rather than a high translation rate. Despite the defects of codon optimization, this strategy is one of the most important methods for improving mRNA translation.

Finally, optimization of the untranslated 5’ UTR and 3’ UTR of mRNA can also significantly improve stability and protein expression (31). For example, start codon AUG or CUG at 5’ UTR can inhibit the translation of mRNA (22). Therefore, avoiding these can improve translation rate of mRNA. Increasing the 3’UTR sequence in tandem may also improve mRNA translation (32). In conclusion, modifications of mRNA structure have proven to be of great importance to improve the stability and protein expression of mRNA vaccines. Future studies will focus on overcoming the limitations of current strategies and developing novel mRNA vaccines with higher stability and translation rate.

## Optimization of the Vectors for Efficient Delivery of mRNA Cancer Vaccines

Despite the many advantages of mRNA cancer vaccine described above, the difficulties of *in vivo* delivery of mRNA vaccine have greatly inhibited its clinical application. Therefore, it is of urgency to increase the delivery efficiency of mRNA vaccines. There are mainly three types of vectors used for *in vivo* delivery: viral, non-viral and cell-based vectors. Dendritic cells (DCs) are usually used as cell-based vectors for adoptive transfer of mRNA cancer vaccines. Designated ‘nature’s adjuvants’, dendritic cells (DCs) are a group of bone marrow-derived antigen-presenting cells that play essential roles in activating and mediating immune response (33, 34). DCs can transit antigen to T cells and facilitate the transduction of immunomodulatory signals *via* cytokines and cell interactions. The generation of mRNA-based cancer vaccines using DCs as vectors involves the use of mRNA extracted from autologous cancers (35). In this process, dendritic cells were isolated from patients, cultured ex vivo, induced to maturation *via* adjuvant, loaded with mRNA encoding target antigens and injected back to patients to activate efficient anti-cancer immunity (36). DC-based mRNA cancer vaccines have been examined by clinical trials (**Table 1**). The initial results from phase I/II have shown that these mRNA vaccines display dramatically increased survival rate and reduced toxicity (37). However, the differentiation, maturation and antigen loading of DCs impact T cell co-stimulation and lead to weak immune response (38, 39). In addition, low production and high variability of mRNA-based DC vaccines have limited the application of this novel immunotherapy. Future studies may focus on the direct injection of mRNA as an alternative approach to overcome the limitations of mRNA-based DC vectors.

**Table 1:** Representative clinical trials of LNP-based and DC-based mRNA cancer vaccines.

Name	RNA encoding antigen	Tumour	Formulation type	Administration route	NCT number	Phase
FixVac	MAGE-A3, NY-ESO-1, tyrosinase, TPTE	Melanoma	LNP	intravenous	NCT02410733	I
mRNA-2416	OX40L	Solid Tumor Malignancies or Lymphoma	LNP	Intratumoural	NCT03323398	I/II
mRNA-2752	OX40L, IL-23, IL-36Ƴ	Solid Tumor Malignancies or Lymphoma	LNP	Intratumoural	NCT03739931	I
mRNA-4157	Personalized neoantigens	Melanoma	LNP	intramuscular	NCT03897881	II
mRNA-4650	Personalized neoantigens	Gastrointestinal cancer	LNP	intramuscular	NCT03480152	I/II
mRNA-5671/V941	KRAS antigens	Colorectal cancer, non-small-cell lung cancer, pancreatic adenocarcinoma	LNP	intramuscular	NCT03948763	I
W_ova1	Ovarian cancer antigens	Ovarian cancer	LNP	intravenous	NCT04163094	I
HARE-40	HPV oncoproteins E6 and E7	HPV oncoproteins E6 and E7	LNP	intradermal	NCT03418480	I/II
RO7198457	Personalized neoantigens	Melanoma	LNP	intravenous	NCT03815058	II
TNBC-MERIT	Personalized neoantigens	Triple-negative breast cancer	LNP	intravenous	NCT02316457	I
MEDI1191	IL-12	Solid tumours	LNP	Intratumoural	NCT03946800	I
SAR441000	IL-12sc, IL-15sushi, IFNα and GM-CSF	Solid tumours	LNP	Intratumoural	NCT03871348	I
TriMixDC-MEL	MAGE-A3, MAGE-C2, tyrosinase, gp100	Melanoma	DC-based	intravenous and intradermal	NCT01066390	I
TriMixDC-MEL	MAGE-A3, MAGE-C2, tyrosinase, gp100	Melanoma	DC-based	intravenous and intradermal	NCT01676779	II
TriMixDC-MEL	CTLA-4 inhibitor ipilimumab	Melanoma	DC-based	intravenous and intradermal	NCT01302496	II
Not available	TAA-transfected DC	melanoma	DC-based	intradermal	NCT01278940	I/II

Viral vectors have been extensively studied for the development of mRNA cancer vaccines, with promising preliminary results being reported (33, 40, 41). For instance, mRNA vaccines for Covid19 pandemic have used viral vectors. In fact, an adenovirus type-5 (Ad5) vector-based RNA vaccines had been on clinical trials in China as early as March 2020 (42). The most used viral vector is self-amplifying mRNA (replicons or saRNA), which encodes target antigen and self-replicates in the cytoplasm of the host cell for expression of target antigen. The viral structural protein is deleted from the sequences of the self-amplifying mRNA thus it is unable to generate infectious virus in the host cells. In the past decades, we have witnessed the utilization of self-amplifying mRNA derived from the genomes of RNA viruses including picornaviruses, alphaviruses and flaviviruses (43, 44). In comparison with non-viral vectors, viral-based mRNA vaccines demonstrate advantages such as modular design, rapid manufacturing and low dose requirement because of its self-replicative properties (45). Viral-based mRNA vaccines against colorectal tumor, urothelial carcinoma, gastroesophageal tumor and rabies have been applied in clinical trials (46). However, the infectious property of virus vectors and the difficulties for large-scale manufacturing of virus vectors have limited the applicability of this approach.

The most famous non-viral vector used for mRNA vaccine is the lipid nanoparticle-based mRNA delivery system, which has been used for the mRNA vaccine of Covid19 and has been very successful (47, 48). LNPs usually consists of an ionizable lipid-like molecule, polyethylene glycol (PEG)-conjugated lipid, cholesterol and a helper phospholipid. Lipid-based nanoparticles (LNPs) could significantly improve delivery efficiency of mRNA cancer vaccines and reduce the system toxicity. Since the ionized lipid is positively charged at low pH, it could enhance the encapsulation of the negatively charged mRNA by electrostatic interaction and promote membrane fusion and destabilization upon delivery. In addition, the neutrality of lipid at the physiological environment improves the stability of mRNA. The first-generation lipids used for LNPs including Dlin-DMA, DLin-KC2-DMA and cKK-E12, etc (49, 50). The second-generation lipids are the derivatives of DLin-KC2-DMA and cKK-E12 (51). In addition to lipid, the other components of LNPs such as cholesterol and phospholipid could facilitate membrane fusion and improve mRNA stability, whereas the diffusive PEG could inhibit particle aggregation, leading to increased stability of mRNA vaccines. Over the past decades, improvements have been made to LPNs. Current LPNs have demonstrated enhanced delivery specificity and improved capacity to be rapidly metabolized and cleared, leading to reduced toxicity of the system and increased stability of mRNA vaccines. Studies have shown that LNPs act as an effective vector for delivery of mRNA vaccines to liver cancers (52, 53). In addition, LPNs have also been reported to target tumours in other organs such as lung, spleen and bone marrow. For example, the Selective Organ Targeting approach developed by Cheng and co-workers using cationic or anionic lipid can target liver, lung and spleen (54, 55). Overall, LNPs have made great contributions to the clinical application of mRNA vaccines.

In addition to LNP vectors, peptide vectors and polymer vectors also play important roles in facilitating the delivery of mRNA cancer vaccines. Polyethylenimine (PEI) is one of the most commonly used polymer-based vectors for mRNA vaccine delivery (56, 57). The efficacy of PEI-based hemagglutinin antigen from influenza virus has been evaluated in mice models (58). In comparison with LNP-based delivery system, polymer vectors display lower purity and reduced clearance rate but higher toxicity. Peptide-based delivery system have been widely studied (59–61). For example, cationic cell-penetrating peptides (CPPs) can condense mRNA complexes and induce strong immune response upon injection (62). However, the precise mechanism of this delivery system remains to be understood.

## Conclusion and Future Perspectives

The successful application of mRNA vaccines for Covid19 has demonstrated the great potential of mRNA vaccines as novel therapies for the treatment of lethal diseases. It’s reported that mRNA-based vaccines can induce immune response in cancer cells, leading to the destruction of cancer cells and the control of tumour growth. Conventional therapies of cancer treatment such as chemotherapy and radiotherapy often suffer from multi-drug resistance and strong toxicity, whereas mRNA-based cancer vaccines have shown enhanced efficacy and reduced toxicity. Therefore, mRNA-based vaccines have gained more and more popularity for the development of novel immunotherapies. However, the instability and *in vivo* delivery of mRNA cancer vaccine have impaired its clinical application. Although progress has been made over the past decades to overcome these limitations, challenges still exist on the development of mRNA cancer vaccines. To promote the wide application of mRNA cancer vaccines, more strategies should be taken to improve the stability and translation rate of mRNA vaccines.

In conclusion, mRNA vaccines have the potential to significantly affect the battle against cancer. Future studies should cast more investigations on the combination of mRNA cancer vaccines and conventional cancer therapies.

## Author Contributions

X-YZ, D-XY and CZ conceived the topic, revised and proofread the manuscript. L-JD and QW drafted the paper and prepared the figure and table. All authors approved the submitted version.

## Funding

This study was supported by the Major Projects in Provincial and National Union Construction of Henan Medical Science Research Plan (SBGJ202001002).

## Conflict of Interest

The authors declare that the research was conducted in the absence of any commercial or financial relationships that could be construed as a potential conflict of interest.

## Publisher’s Note

All claims expressed in this article are solely those of the authors and do not necessarily represent those of their affiliated organizations, or those of the publisher, the editors and the reviewers. Any product that may be evaluated in this article, or claim that may be made by its manufacturer, is not guaranteed or endorsed by the publisher.
